# Transcriptomic data during seed maturation in dormant and non-dormant genotypes of wheat (*Triticum aestivum* L.)

**DOI:** 10.1016/j.dib.2019.104254

**Published:** 2019-07-13

**Authors:** Yuji Yamasaki, Mark C. Jordan, Belay T. Ayele

**Affiliations:** a Department of Plant Science, 222 Agriculture Building, University of Manitoba, Winnipeg, Manitoba R3T 2N2, Canada; b Morden Research and Development Centre, Agriculture and Agri-Food Canada, Morden, Manitoba R6M 1Y5, Canada

**Keywords:** Dormancy, Embryo, Endosperm, Genotype, Microarray, Seed maturation, Wheat

## Abstract

The present data profiles a large scale transcriptome changes in seed tissues (embryo and endosperm) during maturation in dormant and non-dormant genotypes of hexaploid wheat. Seed dormancy is an adaptive trait that has a significant influence on the incidence of preharvest sprouting, which is referred to as the germination of grains on the spike prior to harvest, in wheat. Given that preharvest sprouting causes a substantial yield and quality losses, elucidation of the molecular features that regulate seed dormancy has a paramount significance in the development of preharvest sprouting resistant wheat cultivars. The data presented here was produced from total RNA/mRNA samples isolated from developing seeds of dormant and non-dormant wheat genotypes using the Affymetrix GeneChip Wheat Genome Array. The raw and normalized formats of these data are available in Gene Expression Ominbus (GEO), NCBI's gene expression data repository, with accession number GSE83077.

**Table:** Specifications table

Subject area	Biology
More specific subject area	Transcriptomics of seed maturation
Type of data	Data Table in Excel and.CEL files
How data was acquired	Affymetrix GeneChip Wheat Genome Array
Data format	Raw and normalized data
Experimental factors	Maturing seeds of dormant and non-dormant wheat genotypes
Experimental features	Isolation of total RNA and mRNA, and microarray based large scale transcriptomic analysis.
Data source location	University of Manitoba, Winnipeg, Canada
Data accessibility	Raw and normalized data are available in Gene Expression Ominbus (GEO) database of NCBI with accession number GSE83077
Related research article	Yamasaki et al. [1]. Seed maturation associated transcriptional programs and regulatory networks underlying genotypic difference in seed dormancy and size/weight in wheat (*Triticum aestivum* L.). BMC Plant Biol. 17 (2017) 154, Tuan et al. [2].Transcriptomics of cytokinin and auxin metabolic and signaling genes during seed maturation in dormant and non-dormant wheat genotypes. Sci. Rep. 9 (2019) 3983.

**Table:** 

**Value of the data** • The data profiles tissue specific large scale transcriptome changes during seed maturation in dormant and non-dormant genotypes of wheat. • The transcriptomic data can be used as an important genomic resource for wheat researchers studying transcriptome change in response to loss of dormancy. • The data can be used as a resource to identify genes differentially expressed between different tissues of dormant and non-dormant seeds. • The data is useful to enhance meta-analysis and provides important insights into genes that regulate seed dormancy and thereby preharvest sprouting in wheat.

## 1 Data

**Fig. 1:**
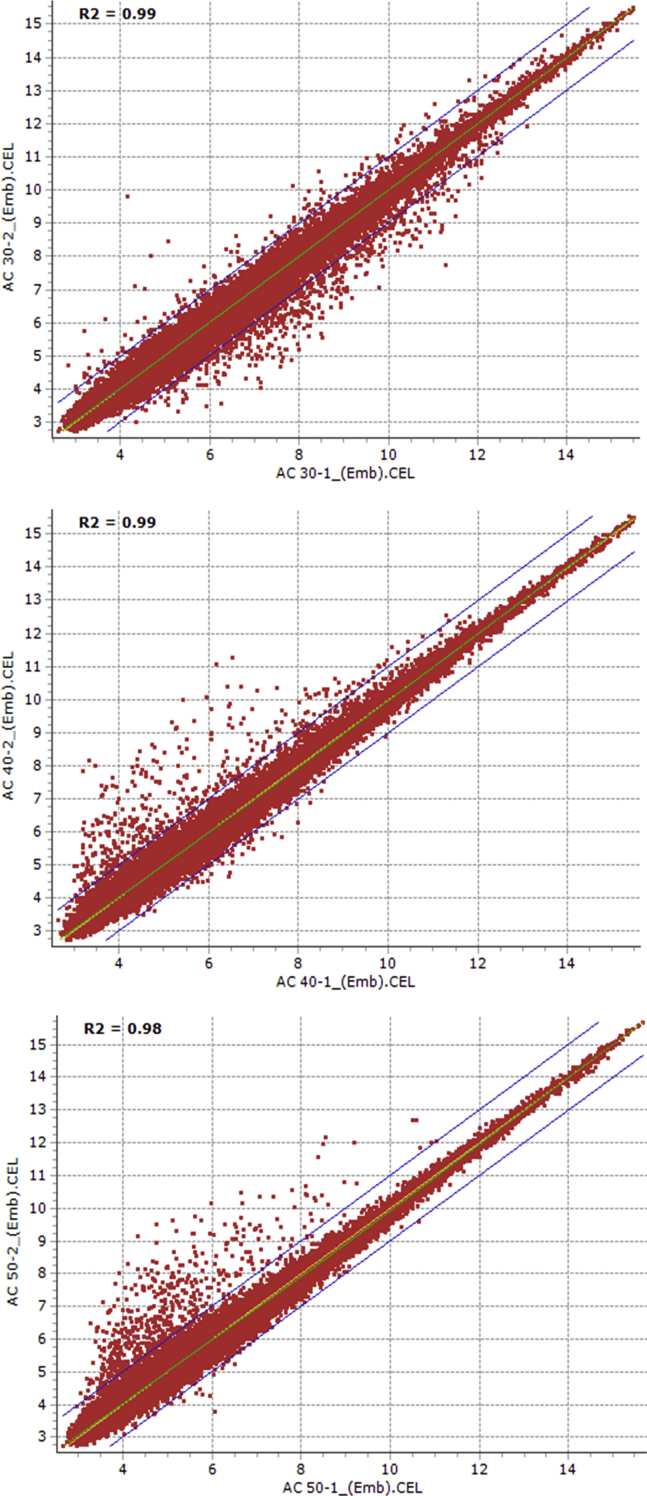
Scatter plots showing the reproducibility of transcriptomic data between replications of 30, 40 and 50 days after anthesis embryo samples of AC Domain genotype.

**Fig. 2:**
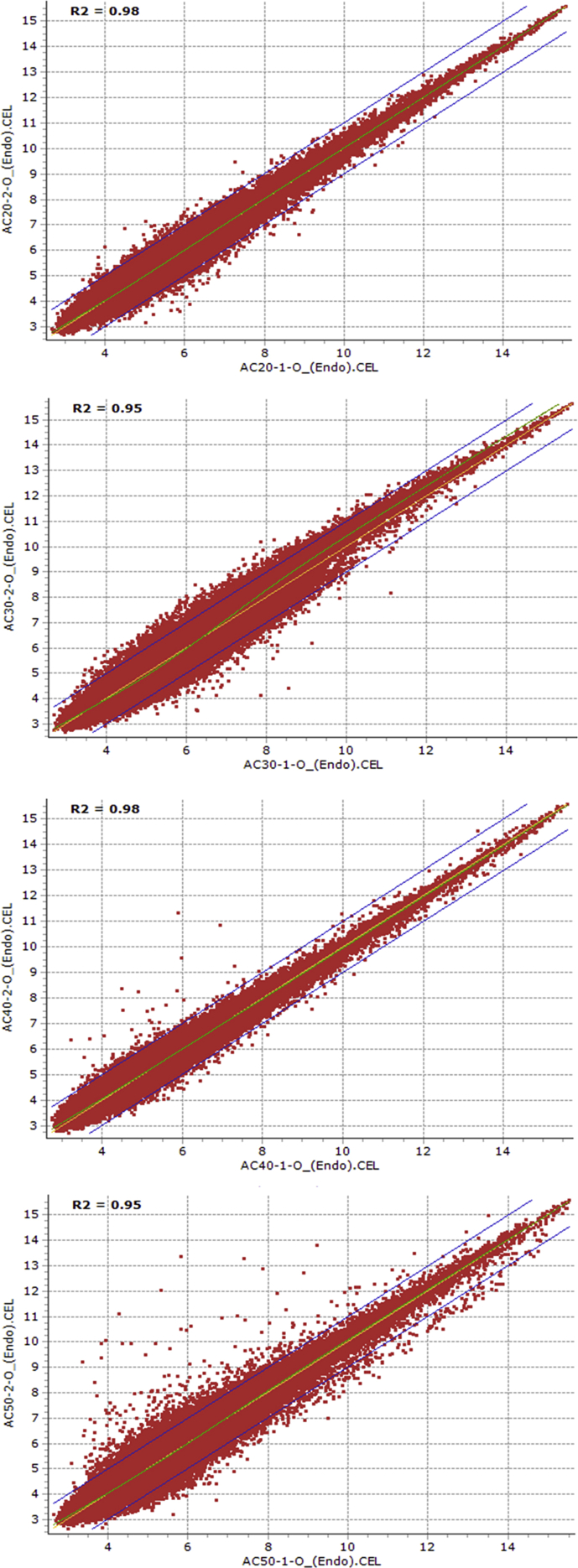
Scatter plots showing the reproducibility of transcriptomic data between replications of 20, 30, 40 and 50 days after anthesis endosperm samples of AC Domain genotype.

**Fig. 3:**
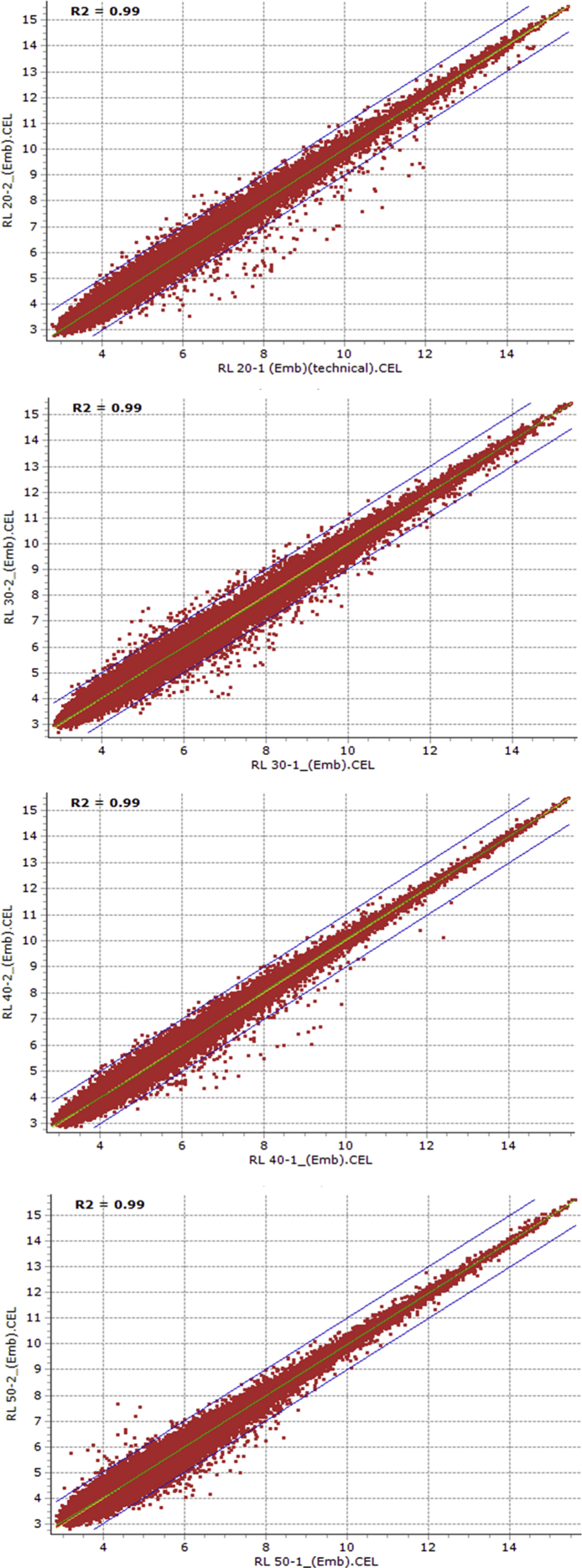
Scatter plots showing the reproducibility of transcriptomic data between replications of 20, 30, 40 and 50 days after anthesis embryo samples of RL4452 genotype.

**Fig. 4:**
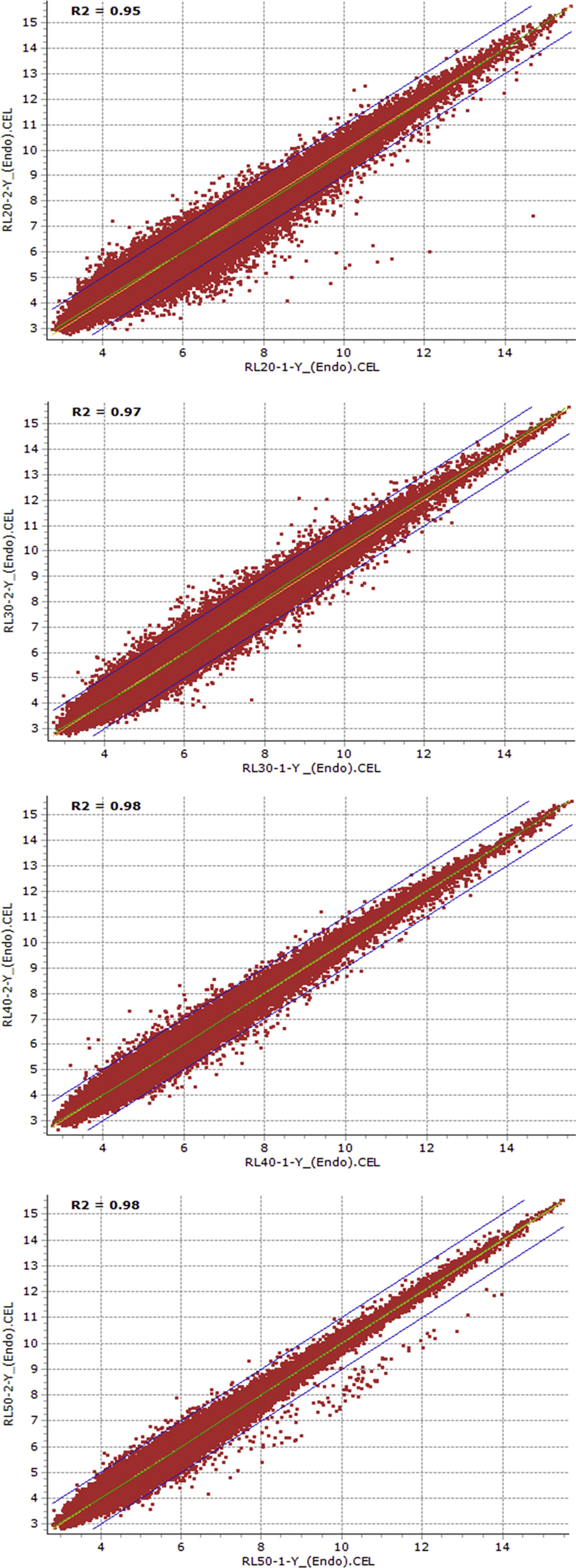
Scatter plots showing the reproducibility of transcriptomic data between replications of 20, 30, 40 and 50 days after anthesis endosperm samples of RL4452 genotype.

This dataset represents large scale transcriptome comparison of embryo and endosperm tissues between dormant and non-dormant wheat genotypes during seed maturation. Total RNA samples were extracted from the embryonic tissues of maturing seeds of the two genotypes while mRNA samples were isolated from the total RNA samples derived from the corresponding endospermic tissues. The total RNA samples of the embryos and mRNA samples of the endosperms of maturing seed samples were subjected to the microarray experiments using Affymetrix GeneChip Wheat Genome Array (Affymetrix, Santa Clara, CA, USA). The raw and normalized formats of these data are available in Gene Expression Ominbus (GEO), NCBI's gene expression data repository (https://www.ncbi.nlm.nih.gov/geo/query/acc.cgi?acc=GSE83077). Reproducibility of the transcriptomic data from the independent replicates of each sample was confirmed by scatter plot expression analysis with the squared Pearson correlation coefficient (R^2^) (Table 1; Fig. 1, Fig. 2, Fig. 3, Fig. 4).

**Table 1:** Reproducibility of transcriptomic data from the independent replicates of 20 DAA embryo samples derived from AC Domain genotype.

Genotype	Embryo	Replicates	R^2^ value
AC Domain	20 DAA^a^	Rep^b^ #1 vs. Rep #2	0.99
		Rep #1 vs. Rep #3	0.99
		Rep #1 vs. Rep #4	0.96
		Rep #2 vs. Rep #3	0.97
		Rep #2 vs. Rep #4	0.97
		Rep #3 vs. Rep #4	0.97

a DAA = days after anthesis.

b Rep = replication.

## 2 Experimental design, materials, and methods

### 2.1 Plant materials and growth conditions

Plant of the dormant wheat genotype AC Domain and the non-dormant genotype RL4452 [3], [4] were grown in a growth chamber at 22 °C/18 °C (day/night) under a 16/8 h photoperiod until harvest as described before [1]. Maturing seeds of each genotype were harvested at different seed maturation stages; from 20 to 50 days after anthesis (DAA). The seed maturation stages studied were determined based on extrusion of the yellow anther in the spikes, which was designated as 0 DAA. Maturing seeds, after harvesting, were separated into embryo (including scutellum) and endosperm (including pericarp and aleurone) tissues and immediately frozen in liquid nitrogen. The seed tissue samples were stored at −80 °C until they were used for RNA isolation.

### 2.2 Isolation of total RNA and mRNA samples

Total RNA was extracted from both embryo and endosperm tissues. The total RNA from the embryos was isolated using RNeasy Plant Mini Kit (Qiagen, Hilden, Germany) while the total RNA from endosperm tissue was isolated as described previously [5]. The total RNA samples from the endosperm tissue were treated with DNase (Ambion, Austin, TX, USA) to remove any contaminating genomic DNA before they are subjected to mRNA isolation using PolyATtract Kit (Promega, Madison, WI, USA) according to the manufacturer's instructions. The microarray experimental procedures of the endosperm tissues were performed with mRNA isolated from the total RNA samples to eliminate the issue of endospermic carbohydrate or starch interference.

### 2.3 Microarray experimental procedure

The total RNA or mRNA samples were used for cDNA synthesis. After purification, biotinylated cRNA samples were prepared using the GeneChip IVT Labelling Kit and the GeneChip Sample Cleanup Module (Affymetrix). Assessment of the quality of the labelled cRNAs was undertaken using an Agilent 2000 Bioanalyzer. Subsequent to fragmentation, labelled cRNA samples were hybridized for 16 hr at 45 °C on GeneChip Wheat Genome Array. Washing and staining of the GeneChips were performed in the Affymetrix Fluidics Station 450. Afterwards, the GeneChips were scanned using an Affymetrix Scanner 3000.

### 2.4 Data analysis

Using the Affymetrix Microarray Suite (MAS5) statistical algorithm, the number of probesets with ‘present’ detection was determined. Subsequently, the raw data was normalized using Robust Multi-array Average (RMA) methodology. HarvEST WheatChip (http://harvest.ucr.edu/) [6] was used to annotate the probesets. The probesets that are differentially expressed between dormant and non-dormant seed tissues were identified using FlexArray software [7] by analysis of variance, and probesets with two or more fold changes at probability level of 0.05 or less were considered differentially expressed. In light of the large number of samples considered and the associated cost, the following experimental strategy was devised to limit the number of replicates and the associated cost without affecting the statistics. Firstly, microarray analysis of the 20 DAA embryo samples of AC Domain genotype was performed using four replicates. Reproducibility of the transcriptome data from any two replicates of the four independent replicates was evaluated using scatter plot expression analysis with the squared Pearson correlation coefficient (R^2^) (Table 1). As a result, microarray analysis of the remaining samples irrespective of genotype, tissue type and maturation stage was performed using two independent replicates, and reproducibility of the transcriptomic data from the two independent replicates of both tissue samples (embryo and endosperm) derived from AC Domain (Fig. 1, Fig. 2) and RL4452 (Fig. 3, Fig. 4) was verified through scatter plot expression analysis as described above.
